# A global land-use data cube 1992–2020 based on the Human Appropriation of Net Primary Production

**DOI:** 10.1038/s41597-025-04788-1

**Published:** 2025-03-27

**Authors:** Sarah Matej, Florian Weidinger, Lisa Kaufmann, Nicolas Roux, Simone Gingrich, Helmut Haberl, Fridolin Krausmann, Karl-Heinz Erb

**Affiliations:** BOKU University Vienna, Institute of Social Ecology, Schottenfeldgasse 29, 1070 Vienna, Austria

**Keywords:** Sustainability, Biodiversity, Environmental impact, Carbon cycle, Agriculture

## Abstract

Land use is intimately linked to key components of the Earth system, including the climate system, biodiversity and biogeochemical cycles. Advanced understanding of patterns and dynamics of land use is vital for assessing impacts on these system components and for developing strategies to ensure sustainability. However, thematically detailed data that enable the analyses of spatiotemporal dynamics of land use, including land-use intensity, are currently lacking. This study presents a comprehensive land-use data cube (LUIcube) that traces global land-use area and intensity developments between 1992 and 2020 annually at 30 arcsecond spatial resolution. It discerns 32 land-use classes that can be aggregated to cropland, grazing land, forestry, built-up land and wilderness. Land-use intensity is represented through the framework of Human Appropriation of Net Primary Production, which allows to quantify changes in NPP, respectively biomass flows, induced by land conversion and land-management. The LUIcube provides the necessary database for analyzing the role of natural and socioeconomic drivers of land-use change and its ecological impacts to inform strategies for sustainable land management.

## Background & Summary

Terrestrial ecosystems provide the basis for human sustenance by supplying food, feed, fuel and fiber, and also play a key role in the global carbon cycle and for biodiversity. With more than two thirds of the global ice-free surface under considerable human use for over one century^1,2^, increasing land-use intensity has become a major factor for resulting pressures and impacts on the functioning of terrestrial ecosystems^3–7^. Addressing the manifold sustainability crises related to biomass use, carbon dynamics, biodiversity and many other ecosystem services hence requires a detailed database on both the extent and intensity of land-use and their dynamics.

Various datasets exist that attempt to capture the extent and intensity of human use of land. Earth observation programs have produced wall-to-wall land cover products in time-series that partially also indicate land uses (such as cropland) over several decades, e.g. the NASA MODIS/Terra + Aqua Land Cover Type classification MCD12Q1v061 from 2001 to 2022^8^, or the ESA Climate Change Initiative land cover product from 1992 onwards^9^. Other remote-sensing based products focus on only one land-cover or use type, such as tree cover development^10^ or cropland patterns and dynamics^11–13^, and hence do not account for the total land area. As the transformation and management of land by humans is not always detectable from space^14^, models like the History Database of the Global Environment (HYDE)^2^ or HILDA+^15^ have additionally integrated census information on land used as cropland or pastures and population density to further progress from the classification of land cover towards land use. Furthermore, several specialized datasets for cropping systems have been developed to provide information on areas used or suitable for the production of certain crops^16–20^, allowing to assess crop-specific impacts on the environment.

The representation and monitoring of land-use intensity is intricate, as the concept is multidimensional and incorporates various components regarding input-, output- or system-level intensity measures^14,21^. Datasets addressing this issue often focus on only one land-use type and specific dimension of land-use intensity, describing intensity through the lens of e.g. irrigation^22,23^, fertilization^24^, livestock density^25,26^, crop yields^17,27,28^ or wood extraction^29,30^.

While these available types of datasets all offer valuable information on certain aspects of the land system, they are limited by one or more of the following shortcomings:

i) An ambiguous differentiation between land cover and land use leads to inconsistent categories within a given dataset and obstructs the attribution of ecosystem impacts to driving processes, like the production of certain goods and services. This particularly affects the differentiation of managed and unmanaged forests or ecosystems that are sporadically used for livestock grazing, which are not addressed in global land-cover products (e.g.^8,9^).
ii) Incomplete area accounts prevail with datasets often representing only one land cover/land use type without offering information on the remaining land area. This impedes the understanding of proximate drivers and trade-offs of land-cover/use change (e.g.^10,12^).
iii) Insufficient thematic detail in land-use types (e.g. regarding cultivars) inhibits the attribution of land system change to production and consumption of specific biomass-based products and related global supply chains (e.g.^1^).
iv) Land-use intensity indicators often represent only the input-, output-, or system-level dimension of the intensity concept and cannot be consistently applied and compared across land-use types. This impedes comprehensive and comparable analyses of land system change^14^ and resulting ecosystem impacts, such as biodiversity^31^.
v) Insufficient spatial, temporal or thematic coverage and resolution restricts analyses of socio-economic or climatic impacts on the patterns and dynamics of the land system.

This study presents the LUIcube (**L**and **U**se **I**ntensity), a novel dataset that overcomes these shortcomings by tracing global land use between 1992 and 2020 annually at 30 arcsecond spatial resolution, consistently discerning 32 land-use classes that can be aggregated to 5 land-use types (cropland, grazing land, forestry, built-up land and wilderness as defined under Methods) and quantifying, for each land-use class, land-use intensity based on the indicator framework Human Appropriation of Net Primary Production (HANPP)^32^. To map land-use areas we advanced established methods^33^ and integrated the land cover product from the European Space Agency Climate Change Initiative^9^ with land-use area information from FAO^34^, the Spatial Production Allocation Model^20^ and other data on human presence^35,36^.

**Fig. 1:**
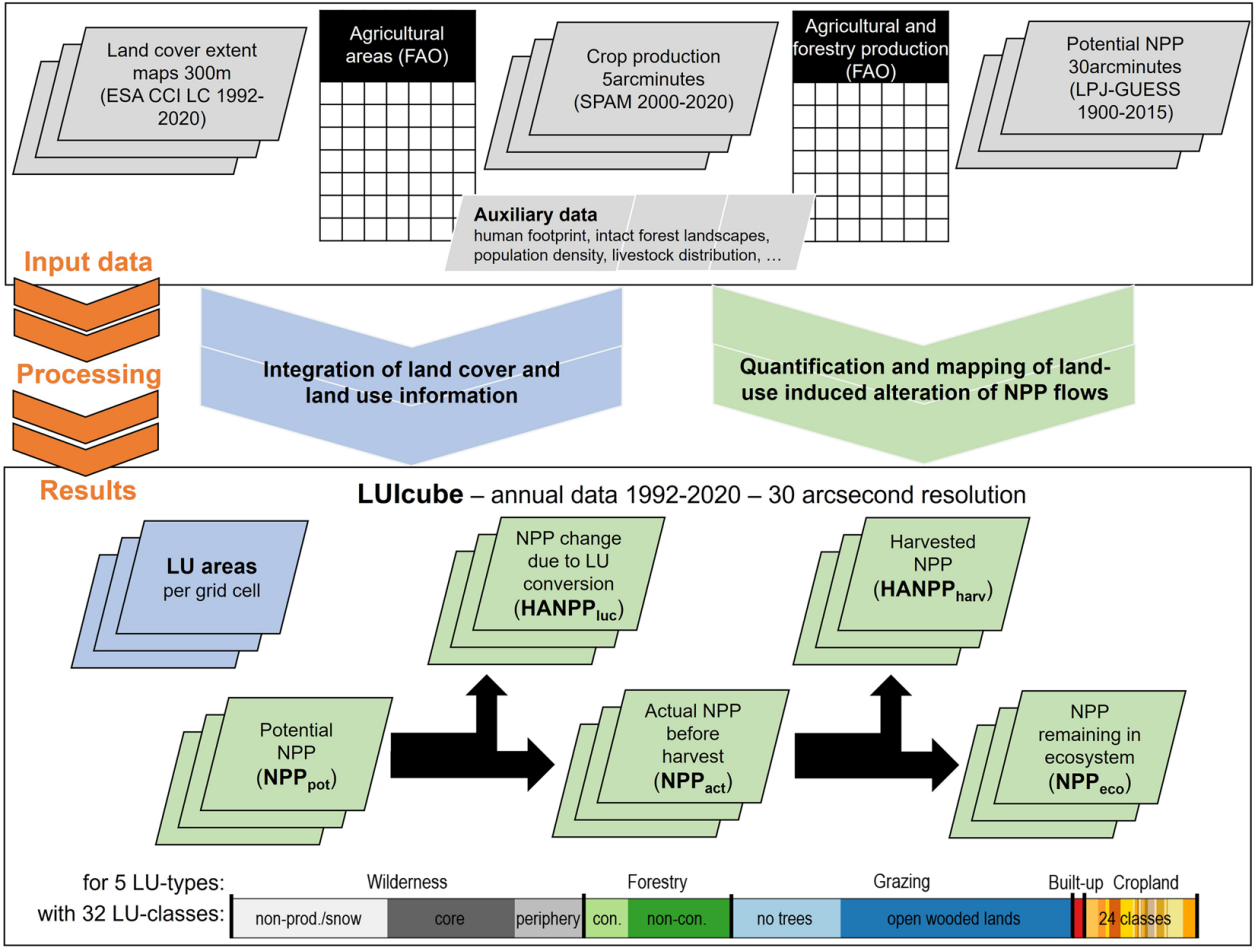
Methodological framework of this study describing the main input datasets, processing steps and results.

To assess land-use intensity, we combined model results on Net Primary Production in the absence of land use (potential NPP, abbreviated NPP_pot_) with agricultural and forestry production statistics from FAO^34^, applying the accounting procedures of the HANPP framework^1,37^ (see Fig. 1 for an overview of the methodological framework, described in detail under Methods).

**Fig. 2:**
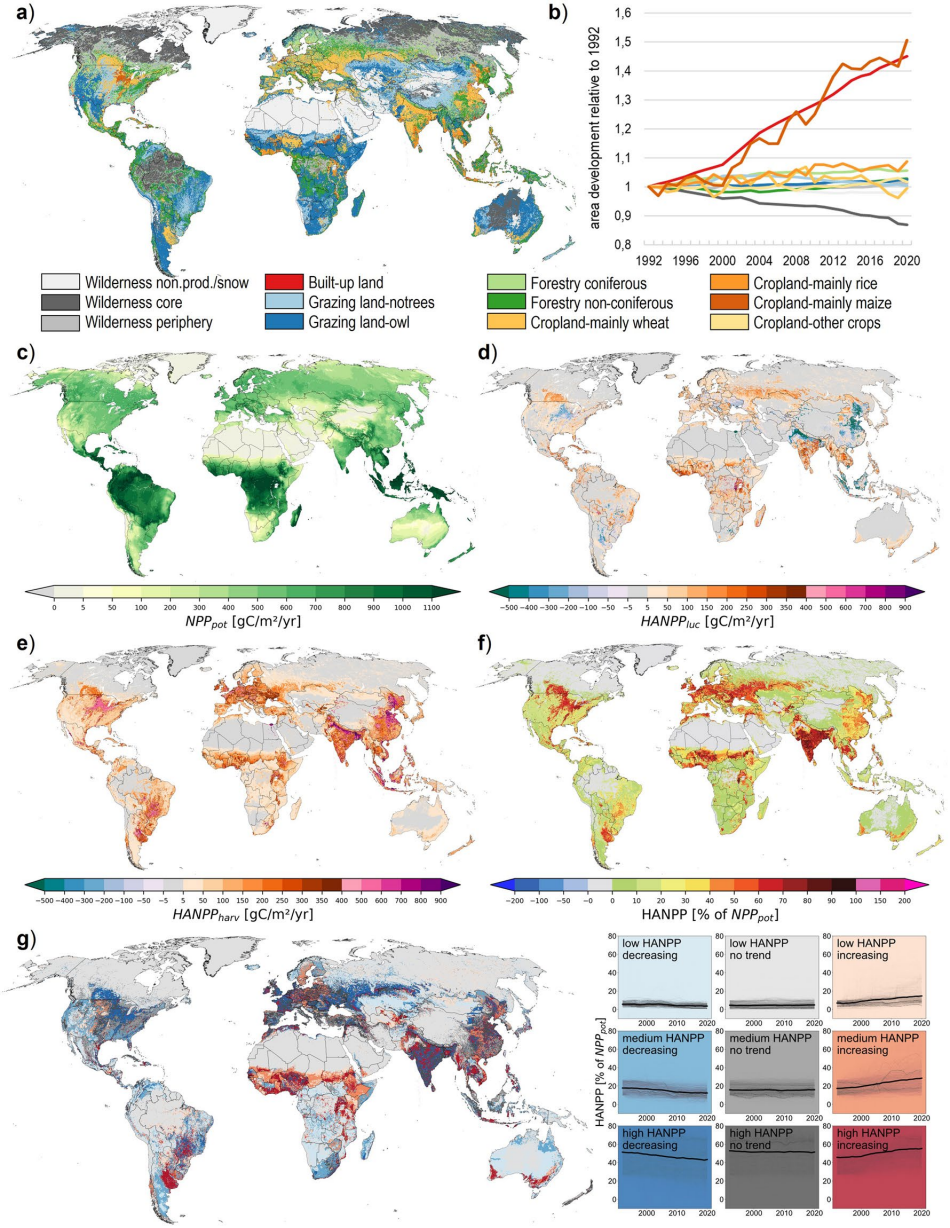
Global pattern of main land-use classes and alteration of NPP flows. The upper panel shows the main land-use class per 30 arcsecond grid cell assigned based on maximum fractional cover for the year 2020 (**a**) and global area development 1992–2020 (**b**). The lower panels present the potential NPP (**c**, NPP_pot_) which is altered through historical land-use conversions (**d**, HANPP_luc_) and extraction of biomass (**e**, HANPP_harv_), for the year 2020. (**d,e**) sum up to total HANPP (**f**). A categorization based on HANPP% level (of 1992; distribution divided into tertiles) and trend 1992–2020 (quantified based on the Theil-Sen estimator^98^ and classified as “decreasing” if below −0.1%/yr, “increasing” if above +0.1%/yr, and as “no trend” if the slope was between these two values) is shown in (**g**).

The LUIcube includes information on area, the change in NPP due to land conversions (HANPP_luc_), the harvested NPP (including losses, HANPP_harv_), and the NPP remaining in ecosystems after harvest (NPP_eco_) for each of the 32 land-use classes in annual time-steps from 1992 to 2020. Adding HANPP_harv_ to NPP_eco_ produces the actual NPP available before harvest (NPP_act_ = NPP_eco_ + HANPP_harv_) and adding HANPP_luc_ to NPP_act_ results in the potential NPP available in the hypothetical absence of land use (NPP_pot_ = NPP_act_ + HANPP_luc_). Global patterns of main land-use classes and the alteration of NPP flows for the year 2020 are shown in Fig. 2.

The consistent quantification of land-use intensity across time, space and products in reference to the key ecosystem parameter Net Primary Production (NPP)^38^ is an important contribution of the LUIcube. The HANPP framework allows to construct land-use intensity indicators integrating dimensions of output intensity (HANPP_harv_) and system-level intensity (HANPP in % of NPP_pot_, NPP_eco_)^21^, which are comparable and applicable across all land-use types. This renders the dataset directly relevant for quantifying, assessing, and understanding human impacts on ecosystem functioning. Altering the NPP available in ecosystems for heterotrophic species is highly pertinent for biodiversity^7,39^ making the LUIcube a valuable resource for biodiversity modelling where spatially explicit information on land-use areas and intensities is critically important^40,41^. Human influence on NPP flows further impacts how much carbon can be stored in ecosystems^4,42,43^.

HANPP_harv_ includes not only the biomass extracted for societal use, but also unused by-products and losses occurring during harvest. This information complements yield assessments necessary for addressing the critical issue of food security and is highly relevant for assessing efficiencies of land and biomass use in general. The LUIcube further allows to investigate to which extent the extraction of biomass follows spatial patterns of natural productivity, or where land conversion results in a reduction or increase in NPP. Although input intensity is not explicitly quantified in the HANPP framework, its effects on NPP flows are indirectly depicted in aggregated terms^44^. For instance, an increase in NPP above potential levels generally indicates the intensive use of inputs like irrigation, fertilizer or pesticides, which adversely affect biodiversity^45^.

**Fig. 3:**
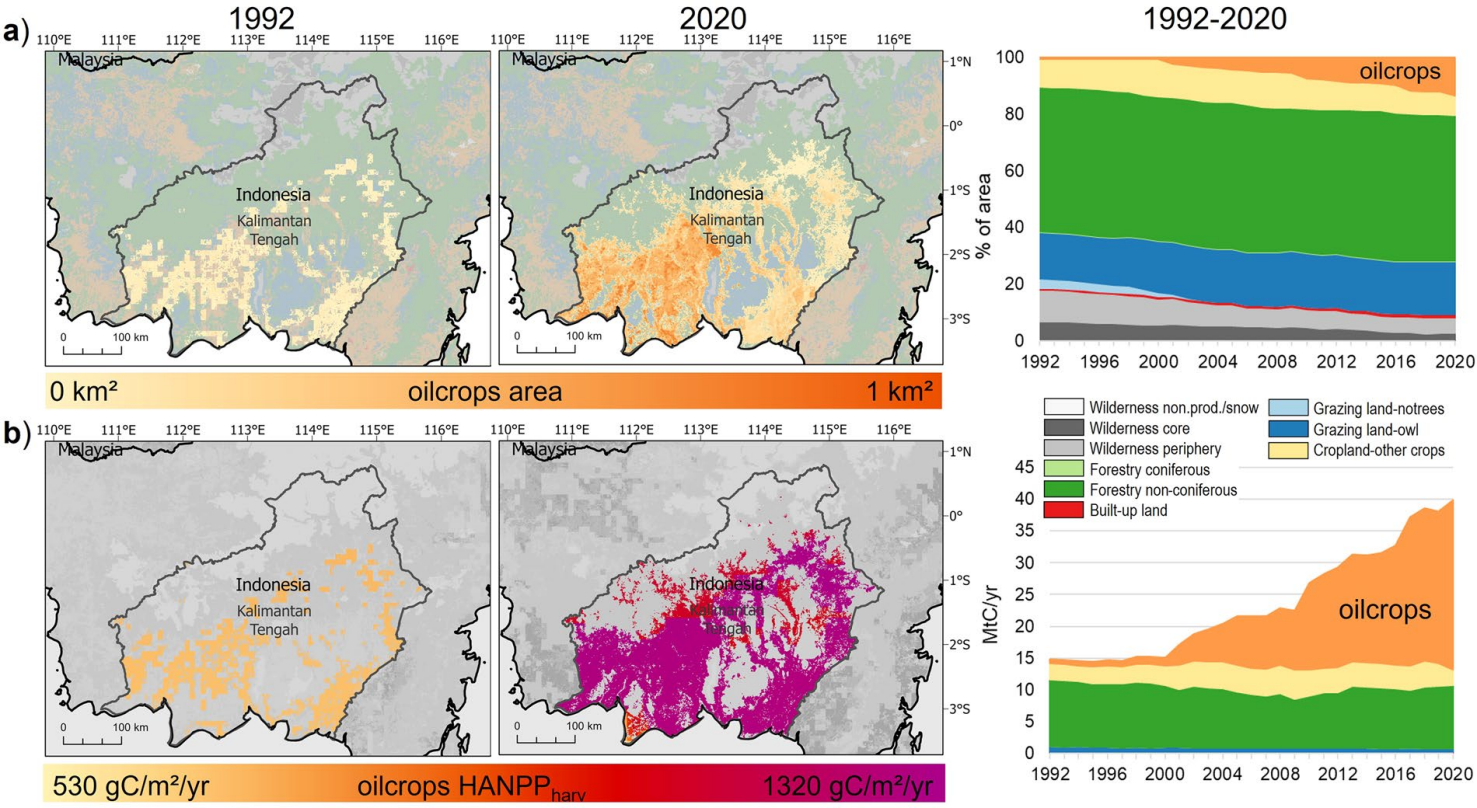
Example of land-use change trajectory: commodity frontier in Kalimantan Tengah (Indonesia) with a focus on oilcrop production. The upper panel shows changes in area (**a**), the lower panel shows changes in HANPP_harv_ (**b**).

**Fig. 4:**
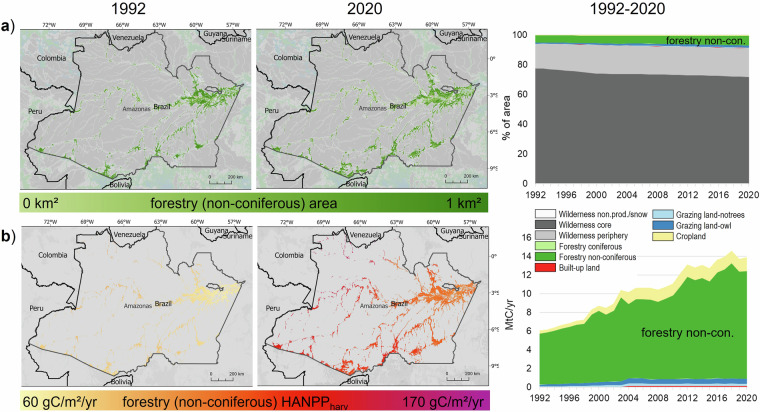
Example of land-use change trajectory: encroachment into wilderness in Amazonas (Brazil) with a focus on non-coniferous forestry. The upper panel shows changes in area (**a**), the lower panel shows changes in HANPP_harv_ (**b**).

**Fig. 5:**
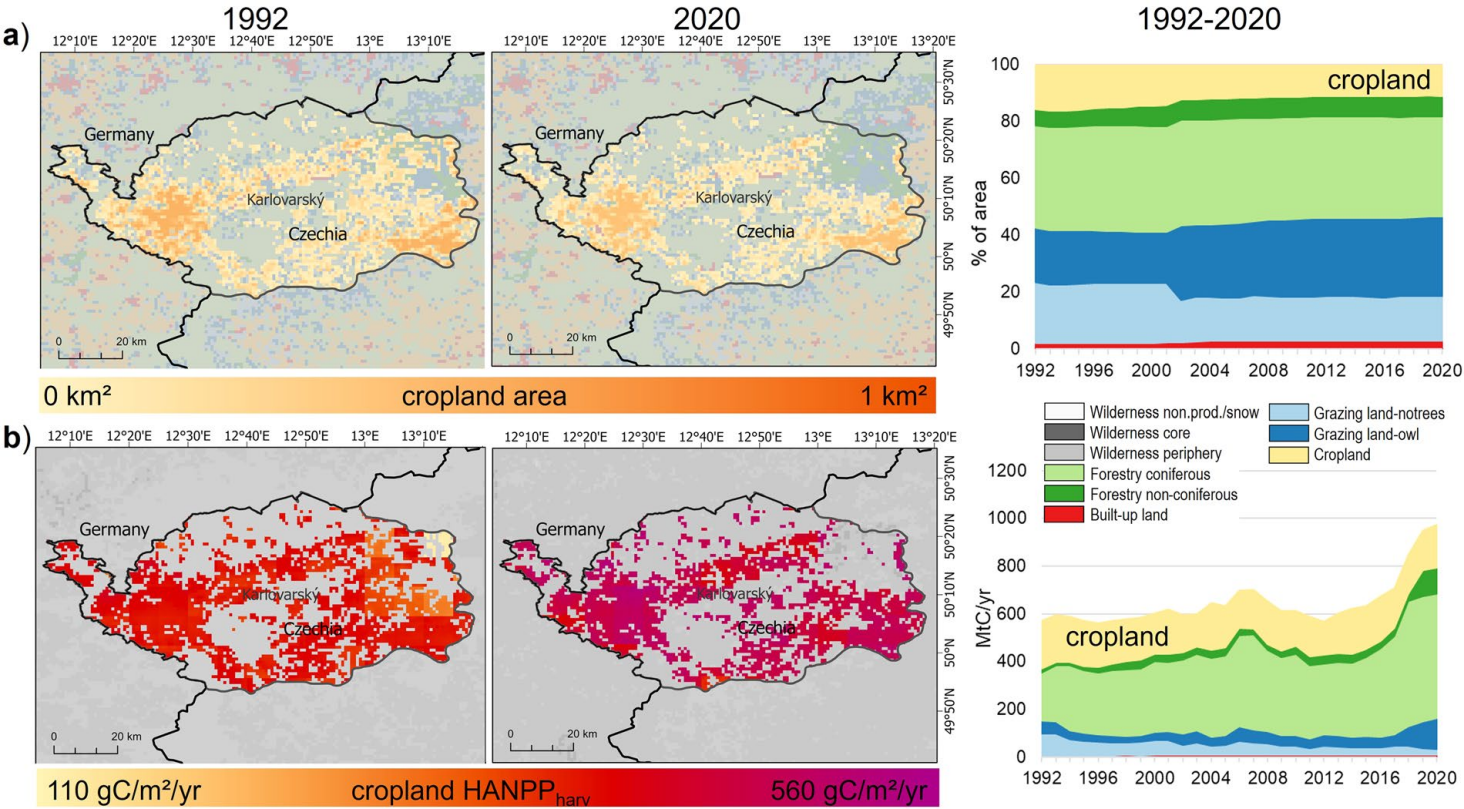
Example of land-use change trajectory: agricultural abandonment in Karlovarsky (Czechia) with a focus on cropland. The upper panel shows changes in area (**a**), the lower panel shows changes in HANPP_harv_ (**b**).

**Fig. 6:**
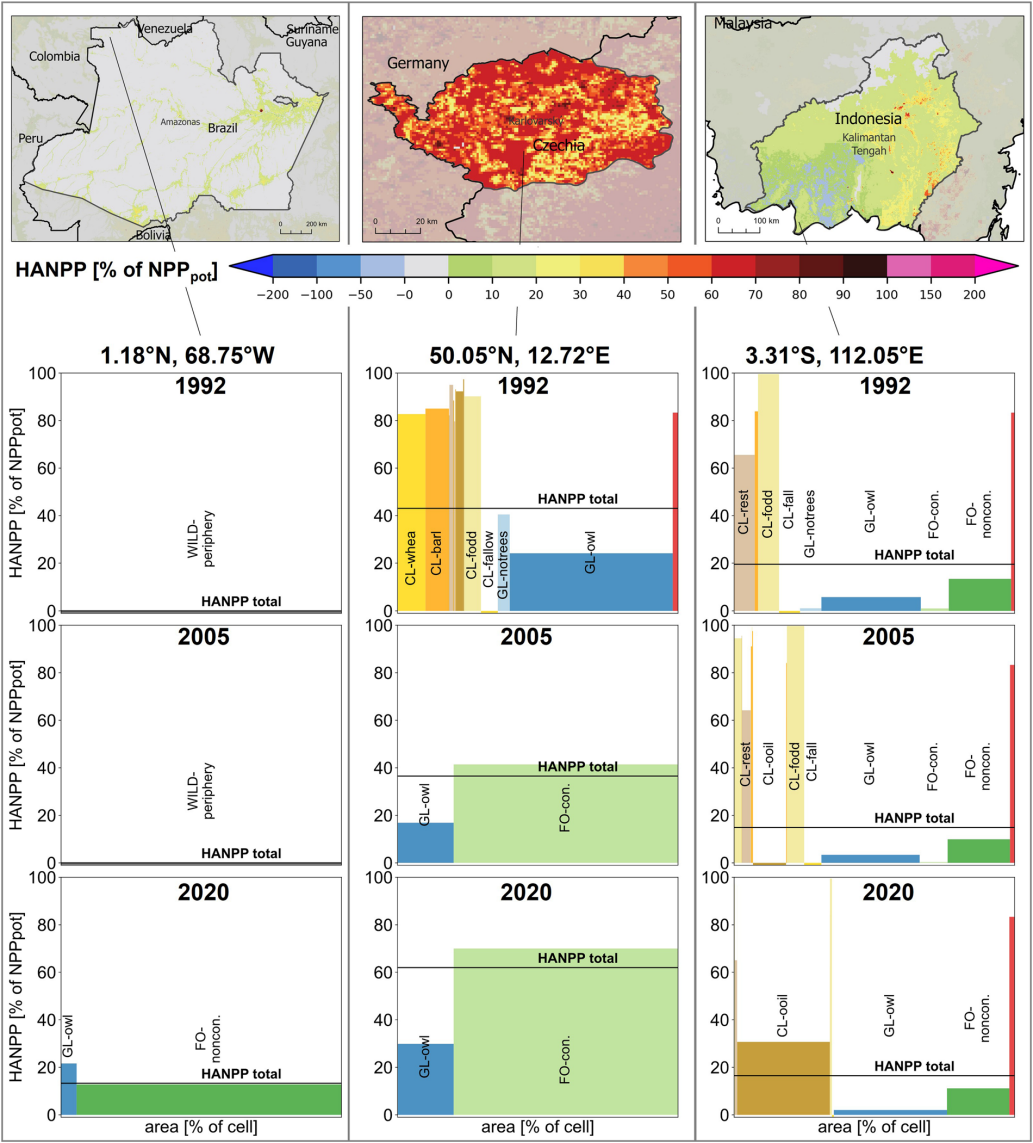
HANPP of selected regions and at grid cell-scale. The upper panel shows the total HANPP as percent of NPP_pot_ for 2020 for Amazonas (Brazil, left), Karlovarsky (Czechia, middle) and Kalimantan Tengah (Indonesia, right), with detailed information per land-use class for one grid cell of the region for 1992, 2005 and 2020 below.

Another asset of the LUIcube is the consistency in methods and data integration over a 29-year time span. This enables the assessment of land-use change trajectories, through the analysis of, for instance, net and gross changes, outliers or inter-annual dynamics at spatially-distinct units (e.g., world regions (see Table 1), biomes, countries, grid cells; see Figs. 3–6). Such applications can address developments like land-use intensification, efficiency change, land-use competition, or polarizations of land use, and lead to the identification of regional or global archetypes of land-use change. In combination with other datasets the LUIcube allows to investigate socio-economic or natural drivers and consequences of land system change and to analyze trade-offs resulting from the ever-increasing demand for land and biomass^46^. Topics for such analyses include the interplay of land-use change with e.g. environmental conflicts^47^, climate extremes^48^, biodiversity^49^, or biogeochemical fluxes^50^. Although the LUIcube focuses on the alteration of carbon flows through continued land use, biomass stock changes due to current land use conversions such as deforestation can also be addressed based on the detailed information on land-use area changes.

**Table 1:** Global and world regional results on land-use areas and HANPP for 2020. Values for wilderness can be derived as the difference between the totals and the four given land-use types.

HANPP 2020		Cropland	Grazing land	Forestry	Built-up	Total	
**Global**	**area** [Mkm^2^]	15.7	47.1	19.9	1.6	**131.6**	
	**NPP**_**pot**_ [MtC/yr]	8 646	23 060	14 510	881	**62 348**	
	**HANPP**_**harv**_ [MtC/yr]	6 776	1 783	1 636	147	**10 342**	
	**HANPP**_**luc**_ [MtC/yr]	−858	1 655	—	588	**1 385**	
	**HANPP** [MtC/yr]	5 918	3 439	1 636	734	**11 727**	
	**HANPP** [% of NPP_pot_]	68%	15%	11%	20%	**19%**	
**Northern America**	**area** [Mkm^2^]	2.3	9.1	3.9	0.3	**28.6**	
	**NPP**_**pot**_ [MtC/yr]	1 121	3 273	2 476	145	**11 187**	
	**HANPP**_**harv**_ [MtC/yr]	875	265	240	24	**1 404**	
	**HANPP**_**luc**_ [MtC/yr]	−88	181	—	96	**190**	
	**HANPP** [MtC/yr]	787	446	240	121	**1 594**	
	**HANPP** [% of NPP_pot_]	70%	14%	10%	20%	**14%**	
**Latin America & the Caribbean**	**area** [Mkm^2^]	1.8	8.4	4.2	0.2	**20.2**	
	**NPP**_**pot**_ [MtC/yr]	1 257	5 196	3 488	119	**15 058**	
	**HANPP**_**harv**_ [MtC/yr]	1 044	439	251	20	**1 754**	
	**HANPP**_**luc**_ [MtC/yr]	−156	366	—	79	**290**	
	**HANPP** [MtC/yr]	888	805	251	99	**2 044**	
	**HANPP** [% of NPP_pot_]	71%	16%	7%	20%	**14%**	
**Central Asia and Russian Federation**	**area** [Mkm^2^]	2.1	5.3	3.4	0.2	**21.4**	
	**NPP**_**pot**_ [MtC/yr]	937	1 973	1 870	85	**9 088**	
	**HANPP**_**harv**_ [MtC/yr]	512	78	126	14	**730**	
	**HANPP**_**luc**_ [MtC/yr]	144	208	—	57	**408**	
	**HANPP** [MtC/yr]	655	285	126	71	**1 138**	
	**HANPP** [% of NPP_pot_]	70%	14%	7%	20%	**13%**	
**Europe**	**area** [Mkm^2^]	1.2	1.8	1.4	0.2	**4.7**	
	**NPP**_**pot**_ [MtC/yr]	637	999	841	112	**2 681**	
	**HANPP**_**harv**_ [MtC/yr]	501	114	182	19	**816**	
	**HANPP**_**luc**_ [MtC/yr]	−51	134	—	74	**158**	
	**HANPP** [MtC/yr]	451	248	182	93	**974**	
	**HANPP** [% of NPP_pot_]	71%	25%	22%	20%	**36%**	
**Africa**	**area** [Mkm^2^]	3.3	14.1	3.0	0.3	**34.1**	
	**NPP**_**pot**_ [MtC/yr]	1 879	7 458	2 738	137	**13 412**	
	**HANPP**_**harv**_ [MtC/yr]	835	652	196	23	**1 707**	
	**HANPP**_**luc**_ [MtC/yr]	517	378	—	91	**986**	
	**HANPP** [MtC/yr]	1 352	1 030	196	114	**2 692**	
	**HANPP** [% of NPP_pot_]	72%	14%	7%	20%	**20%**	
**Asia**	**area** [Mkm^2^]	4.9	8.5	4.0	0.5	**22.5**	
	**NPP**_**pot**_ [MtC/yr]	2 815	4 162	3 096	284	**10 922**	
	**HANPP**_**harv**_ [MtC/yr]	3 009	235	639	47	**3 931**	
	**HANPP**_**luc**_ [MtC/yr]	−1 224	388	—	190	**−646**	
	**HANPP** [MtC/yr]	1 786	623	639	237	**3 285**	
	**HANPP** [% of NPP_pot_]	63%	15%	21%	20%	**30%**

The high thematic resolution of the LUIcube further enables investigating the role of specific crops or other biomass products in spatial patterns, temporal dynamics or ecological impacts of human land use, and can provide important context to studies of, for example, agricultural commodity frontiers (see Fig. 3), or agricultural abandonment (see Fig. 5). Such spatiotemporal analyses of land-use intensity change enabled by the HANPP framework not only allow for a better understanding of land-use dynamics and patterns, but can also inform the model-based assessments of future scenarios through calibration or parametrization. These versatile possible applications of the LUIcube are helpful in advancing the understanding of the intricate drivers, processes and consequences of land-use.

## Methods

The LUIcube presents information on area and biomass flows for 32 land-use classes globally at 30 arcsecond resolution and in annual time steps from 1992 to 2020. It was constructed based on the datasets listed in Table 2 and advancements of methods described in^1,51^.

**Table 2:** List of datasets and download links used in this study.

Dataset name and citation	Link for data access	Use in this study
ESA CCI LC^95^	https://cds.climate.copernicus.eu/datasets/satellite-land-cover?tab=download	Allocation of land-use types to the 30 arcsecond grid
SPAM 2000^58^	10.7910/DVN/A50I2T	Allocation of crop (class)-specific production patterns; based on harvested and physical area and yield
SPAM 2005^59^	10.7910/DVN/DHXBJX	
SPAM 2010^60^	10.7910/DVN/PRFF8V	
SPAM 2020^61^	10.7910/DVN/SWPENT	
Human footprint 2000-2018^65^	10.6084/m9.figshare.16571064	Identification of wilderness and unused areas
Human footprint 1993^96^	https://datadryad.org/stash/dataset/doi:10.5061/dryad.052q5	
Intact forest landscapes^64^	https://intactforests.org/data.ifl.html	
Gridded livestock of the world 2010^61^	https://dataverse.harvard.edu/dataverse/gld	Assessment of influence of population density on ruminant livestock distribution
Global human settlement layer^80^	https://jeodpp.jrc.ec.europa.eu/ftp/jrc-opendata/GHSL/GHS_POP_GLOBE_R2023A/	
NPP_pot_ from LPJ-GUESS without nitrogen limitation^97^	10.5281/zenodo.5519104	Downscaled to 30 arcseconds based on^70^; used as reference point for HANPP calculation
FAOstat^34^ Land Use	https://www.fao.org/faostat/en/#data/RL items: cropland, permanent meadows and pastures download date: 22^nd^ May of 2024	Target value for cropland and grazing land areas per country
FAOstat^34^ Crops and livestock products	https://www.fao.org/faostat/en/#data/QCL items: crops production, area harvested items: livestock production download date: 10^th^ June of 2024	Target value for areas harvested per crop and primary biomass harvest as basis for HANPP_harv_ and NPP_act_ calculation
FAOstat^34^ Forestry production and Trade	https://www.fao.org/faostat/en/#data/FO items: production quantity of wood fuel and industrial roundwood download date: 10^th^ June of 2024	Primary wood extraction as basis for HANPP_harv_ calculation for forestry and open wooded lands
FAOstat^34^ Food Balances, Supply Utilization Accounts	https://www.fao.org/faostat/en/#data/SCL download date: 10^th^ June of 2024	Input for calculating livestock feed supplied by markets to calculate grazing demand
FAOstat^34^ Bilateral Trade matrices	https://www.fao.org/faostat/en/#data/TM download date: 10^th^ June of 2024	

### Land-use areas

The land-use area dataset was constructed based on a closed-budget approach^33^, assigning the total land area to 5 main land-use types, that are further differentiated into 32 land-use classes.

We primarily used the land cover product from the European Space Agency Climate Change Initiative (ESA CCI LC)^9,52^ to allocate the land-use types to the grid by defining the correspondence between land cover and land use (Table 3) based on the suitability of certain land-cover classes for a given land use. For cropland and grazing land national area statistics are available from FAOstat^34^. Here, we used these suitability classes to distribute the national totals sequentially, starting with the most suitable class. Non-productive areas (including snow) were mapped based on the potential productivity and other wilderness areas were derived from^35,36^ (see below for details).

**Table 3:** Suitability of ESA CCI land cover classes for land-use types constructed in the LUIcube.

ESA CCI LC map description	LUIcube – land-use types										
	Built-up land	Cropland suitability class1	Cropland suitability class2	Cropland suitability class3	Grazing suitability class1	Grazing suitability class2	Grazing suitability class3	Closed Forest – coniferous	Closed Forest –non-coniferous	Open wooded land – coniferous	Open wooded land –non-coniferous
Cropland, rainfed		95			100						
Herbaceous cover		95			100						
Tree or shrub cover		95			100						
Cropland, irrigated or post-flooding		95			100						
Mosaic cropland (>50%) / natural vegetation (tree, shrub, herbaceous cover) (<50%)		60			60	40				8	32
Mosaic natural vegetation (tree, shrub, herbaceous cover) (>50%)/cropland (<50%)		40			40	60				13	47
Tree cover, broadleaved, evergreen, closed to open (>15%)									100		
Tree cover, broadleaved, deciduous, closed to open (>15%)									100		
Tree cover, broadleaved, deciduous, closed (>40%)									100		
Tree cover, broadleaved, deciduous, open (15–40%)			35			100					55
Tree cover, needleleaved, evergreen, closed to open (>15%)								100			
Tree cover, needleleaved, evergreen, closed (>40%)								100			
Tree cover, needleleaved, evergreen, open (15–40%)			30			100				35	5
Tree cover, needleleaved, deciduous, closed to open (>15%)								100			
Tree cover, needleleaved, deciduous, closed (>40%)								100			
Tree cover, needleleaved, deciduous, open (15–40%)			30			100				35	5
Tree cover, mixed leaf type (broadleaved and needleleaved)								50	50		
Mosaic tree and shrub (>50%) / herbaceous cover (<50%)			40			100				15	45
Mosaic herbaceous cover (>50%) / tree and shrub (<50%)			60			100				10	30
Shrubland			95			100				20	40
Evergreen shrubland			95			100				30	30
Deciduous shrubland			95			100					60
Grassland			95		100						
Lichens and mosses							100				
Sparse vegetation (tree, shrub, herbaceous cover) (<15%)				95			100				
Sparse tree (<15%)				95			100			2	8
Sparse shrub (<15%)				95			100			2	8
Sparse herbaceous cover (<15%)				95			100				
Tree cover, flooded, fresh or brakish water								50	50		
Tree cover, flooded, saline water									100		
Shrub or herbaceous cover, flooded, fresh/saline/brakish water							100				
Urban areas	100										

The share of area (given in %) per ESA land cover class suitable for each land-use type is based on the represented plant functional types^9^ and provides the basis for the integration of national level census data on land-use areas. During the sequential land-use type allocation, overlapping suitability shares are reduced by subtracting the already allocated area.

**Fig. 7:**
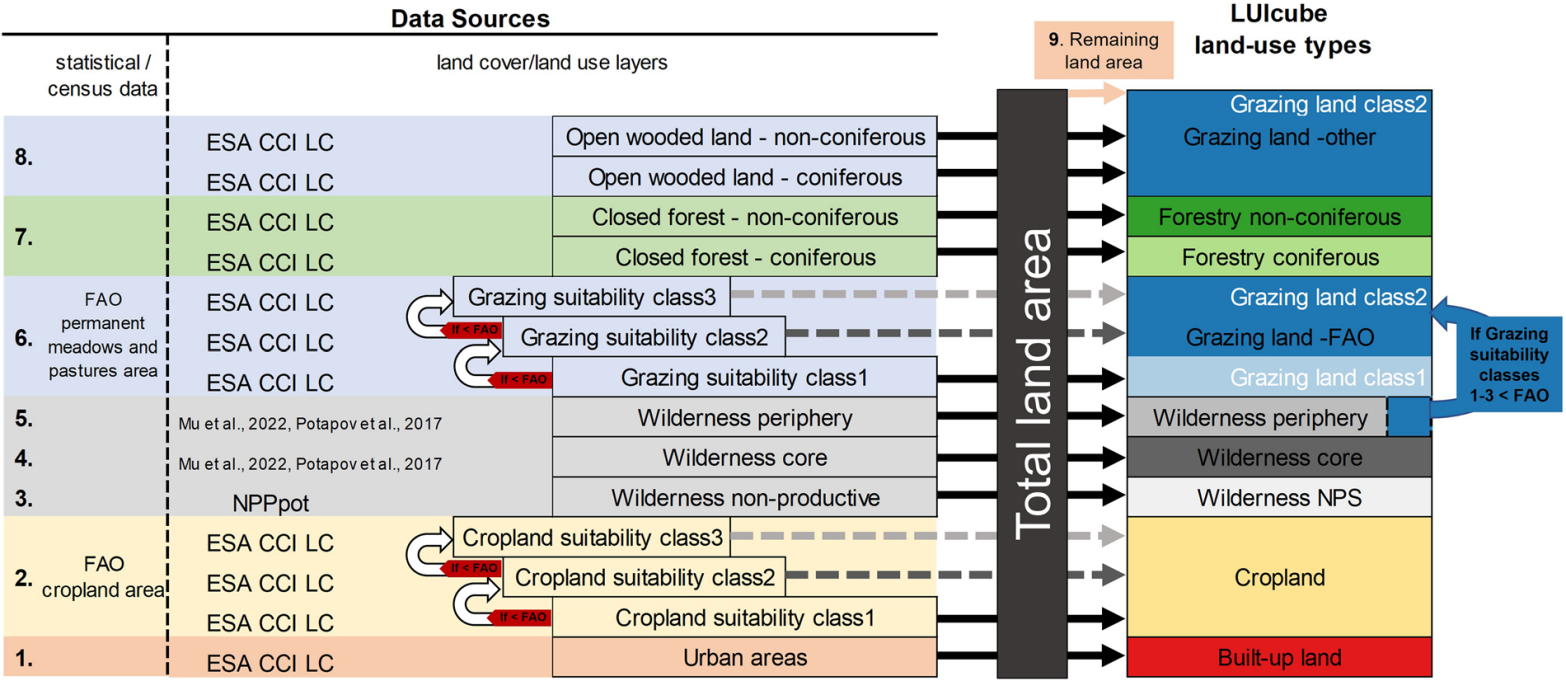
Stepwise integration of data inputs for mapping land-use types.

Steps 1 through 9 (as shown in Fig. 7) were conducted in sequence, filling up the total land area per grid cell.

First, we mapped built-up land based on urban areas from ESA CCI LC. Next, the national cropland areas from FAO were allocated to the cropland suitability classes 1 to 3 sequentially. Cropland was then further differentiated into 23 crop-specific LU classes and fallow land, mainly based on the Spatial Production Allocation Model^20^. Non-productive wilderness was mapped based on the potential productivity, using a threshold of 20gC/m²/yr for NPP_pot_^33^. Next, wilderness core and periphery were assigned. As a 6^th^ step, the national totals for permanent meadows and pastures reported in FAOstat were distributed within grazing suitability classes 1 to 3 per country. Since the grazing suitability classes 1 and 2 overlap with the same ESA CCI LC classes as cropland suitability classes 1 and 2, the assigned cropland areas were subtracted before distributing the national FAO total for grazing land. If the remainder of the grazing suitability classes 1 to 3 was smaller than the FAO value, part of peripheral wilderness was reclassified as grazing land. In step 7, forestry was mapped based on the ESA CCI LC classes for closed forests. Remaining open wooded land (step 8), comprising open forests with tree cover below 40% and the remaining land area not accounted for in the preceding steps were categorized as other grazing land (additionally to the FAO grazing land total). These steps are described in detail below and a full list of all LU classes is presented in Table 6.

#### Land cover product

The compilation of the land-use dataset aimed to reassemble land-cover data available from remote-sensing based on information on how this land is used by humans. While several global land-cover products are available (e.g.^53,54^), the ESA CCI land cover product was selected as it provides the longest annual time series starting in 1992. We also chose not to combine multiple land cover products to maintain simplicity and facilitate seamless integration with other ESA products. We defined the correspondence between land cover and land use based on the plant functional types of each land cover class (see Table 3). The ESA CCI LC product was resampled from the original resolution of 300 m to fractional cover at 30 arcseconds, which is approximately 1 km^2^ at the equator, and consists of 9 grid cells from the original resolution. This resolution was chosen (1) to reduce computational requirements, (2) to match the resolution of the change detection module of the ESA CCI LC product^9^, and (3) because it was found to be an appropriate resolution to link HANPP and biodiversity^55^.

#### Land area and coastlines

The total land area per 30 arcsecond grid cell was calculated by subtracting ESA CCI LC class “210-Water bodies” from the total area of the grid cell. This was the starting point for the distribution of the various land-use classes within each cell.

#### Built-up land

Built-up land was mapped using the ESA CCI LC class “190-Urban areas”. These areas are not completely sealed, but also include parks or gardens. Additionally, we allocated 5% of the cropland area in each grid cell to built-up land to account for dispersed settlements and infrastructure not captured at the ESA CCI LC resolution of 300 m.

#### Cropland

The aim of the LUIcube is to track changes in cropland area and patterns across time, warranting consistency with national cropland totals reported by FAO. We thus used year-specific cropland patterns derived from ESA CCI LC adjusted to match the reported national cropland areas from FAOstat.

Data on the total area used as cropland per country^56^ in addition to information on crop-specific area harvested per year^57^ were downloaded from FAOstat on 22^nd^ May of 2024.

We classified several ESA CCI LC classes as being potentially used as cropland (see Table 3), with decreasing suitability from class1 to class3 and distributed the national total given by FAO (CLarea_FAO_) consecutively across these classes in following procedure, which in principle follows the one suggested by^2^:

If the national total of cropland suitability class1, derived from ESA CCI LC, exceeded CLarea_FAO_, class1 cells were assigned to cropland proportionally, with the remainder per cell being available for grazing. If not, all class1 cells were used as cropland. If the remaining area of CLarea_FAO_ of the country fit into cropland suitability class2 cells, it was distributed in the direct proximity of class1 cells. If not, cropland suitability class3 was utilized in the same manner. The national cropland area was reduced if the sum of suitability class1, class2 and class3 could not cover the value reported by FAOstat.

To differentiate cropland into the production areas for specific crops or crop classes we used data from the Spatial Production Allocation Model (SPAM)^20,28^ for 2000^58^, 2005^59^, 2010^60^ and 2020^61^, given at a spatial resolution of 5 arcminutes. This dataset is constructed based on subnational statistical data on crop production and uses auxiliary information on crop prices, population density and biophysical suitability to distribute cropping systems across grid cells. Since the thematic resolution was increased over time, we created aggregated crop classes (Table 4) to work with a continuous time series.

**Table 4:** Aggregation of SPAM categories and allocation of FAO primary crops to the corresponding 22 food crops/crop classes in the LUIcube.

LU class	LU class description	FAO code	SPAM2000	SPAM2005, SPAM2010	SPAM2020
CL-BANP	banana / plantain	486	BANP	BANA	BANA
		489	BANP	PLNT	PLNT
CL-BARL	barley	44	BARL	BARL	BARL
CL-BEAN	bean	176	BEAN	BEAN	BEAN
CL-CASS	cassava	125	CASS	CASS	CASS
CL-COFF	coffee	656	COFF	ACOF, RFOC	ACOF, RCOF
CL-COTT	cotton	328	COTT	COTT	COTT
CL-GROU	groundnut	242	GROU	GROU	GROU
CL-MAIZ	maize	56	MAIZ	MAIZ	MAIZ
CL-MILL	millet	79	MILL	PMIL, SMIL	PMIL, SMIL
CL-OFIB	other fiber crops	773, 777, 780, 782, 788, 789, 800, 809, 813, 821	OFIB	OFIB	OFIB
CL-OOIL	other oil crops	260, 263, 292, 265, 275, 277, 280, 296, 299, 305, 310, 333, 339, 336	OOIL	OOIL	OOIL
		249	OOIL	CNUT	CNUT
		254	OOIL	OILP	OILP
		267	OOIL	SUNF	SUNF
		270	OOIL	RAPE	RAPE
		289	OOIL	SESA	SESA
CL-OOIL	other pulses	181, 187, 203, 205, 210, 211	OPUL	OPUL	OPUL
		191	OPUL	CHIC	CHIC
		195	OPUL	COWP	COWP
		197	OPUL	PIGE	PIGE
		201	OPUL	LENT	LENT
CL-POTA	potato	116	POTA	POTA	POTA
CL-REST	rest of crops	71, 75, 89, 92, 94, 97, 101, 103, 108	OTHE	OCER	OCER
		135, 136, 149	OTHE	ORTS	ORTS
		161, 216, 217, 220, 221, 222, 223, 224, 225, 226, 234, 671, 677, 687, 689, 692, 693, 698, 702, 711, 720, 723, 748, 754	OTHE	REST	REST
		661	OTHE	COCO	COCO
		667	OTHE	TEAS	TEAS
		826	OTHE	TOBA	TOBA
		836	OTHE	REST	RUBB
CL-RICE	rice	27	RICE	RICE	RICE
CL-SORG	sorghum	83	SORG	SORG	SORG
CL-SOYB	soybean	236	SOYB	SOYB	SOYB
CL-SUGB	sugar beet	157	SUGB	SUGB	SUGB
CL-SUGC	sugarcane	156	SUGC	SUGC	SUGC
CL-SWPY	sweet potato and yam	122	SWPY	SWPO	SWPO
		137	SWPY	YAMS	YAMS
CL-VEFR	vegetables / fruits	358, 366, 367, 372, 373, 378, 393, 394, 397, 399, 401, 406, 407, 414, 417, 420, 423, 426, 430, 446, 449, 459, 461, 463	OTHE	VEGE	VEGE
		567, 568, 569, 571, 572, 574, 577, 587, 591, 600, 603	OTHE	TROF	TROF
		515, 521, 523, 526, 530, 531, 534, 536, 541, 542, 544, 547, 549, 550, 552, 554, 558, 560, 619	OTHE	TEMF	TEMF
		388	OTHE	VEGE	TOMA
		402, 403	OTHE	VEGE	ONIO
		490, 495, 497, 507, 512	OTHE	VEGE	CITR
CL-WHEA	wheat	15	WHEA	WHEA	WHEA

For each crop, SPAM provides information on yield, harvested area, and physical area accounting for multicropping within one year. For each gird cell we interpolated linearly between years with data points and kept values constant before the first data point (i.e., 1992–2000). The data obtained this way was integrated with the total cropland area as follows:

The basis cropland map used in SPAM is a synergy map^62^ that combines multiple sources to arrive at a cropland map for circa 2010. While this approach might create a more accurate cropland map for this point in time, tracking changes in cropland area and patterns across time requires using year-specific cropland patterns, which we derived from ESA CCI LC and adjusted to match the reported cropland areas from FAO per country as described above. Hence, we adapted the SPAM layers for harvested area per crop to the LUIcube cropland map of each year. This was done by filling those cropland cells not covered by the SPAM synergy cropland map with the respective average value of harvested area per crop of the closest cells in a radius of 30 cells (~30 km) and omitting the SPAM information of cells not classified as cropland in the LUIcube.

We ensured consistency with the harvested areas reported by FAO through following procedure:

1. The minimum fallow area per country was estimated by relating the total harvested area (CLharv_FAO_), summed over the 22 crop classes *c* derived from SPAM, to the total cropland area at the national level (*Clarea*_*FAO*_), and applying the resulting percentage to the cropland area of each cell to arrive at the area per cell that is available for harvesting (CLharv).
  1 $$FALLOWmi{n}_{FAO}=CLare{a}_{FAO}-\mathop{\sum }\limits_{c=1}^{22}AREAharveste{d}_{FAO,c}$$
2. The area available for harvesting (CLharv) was then distributed among the 22 crop classes based on the share of each crop’s physical area in the sum of the physical areas of all crops present per cell (S_area_c_) according to the SPAM dataset (adjusted to the cropland pattern and extent of the given year). The resulting crop-specific cropland area per cell (CLharv_c_) was then adjusted based on a factor derived on the national level to match the FAO harvested area given for this crop and country (f_c, FAO_). If a crop in a country was not represented in SPAM, but was reported in FAO we assumed it to be cropped proportionally in all cropland cells of the country.
  2 $$CLhar{v}_{c,FAO}=(CLharv\ast S\_are{a}_{c})\ast {f}_{c,FAO}$$
3. Applying the multicropping index given in SPAM to CLharv_c,FAO_, we then arrived at the physical area per crop. Due to multicropping, the physical area can be smaller than the harvested area, which would increase the initial fallow areas estimate from step 1.
4. While food crops are not cultivated on this fallow area, we assigned the production of fodder crops, for which data is not reported in the FAO database, to 50% of this area. The other 50% were assumed to actually lay fallow.

The multicropping index in cropland cells not identified as such in SPAM was set to the median value of the country. We used the global median value of the respective crop class if no SPAM data for the given country was available.

#### Wilderness

The land-use type wilderness was defined as areas that are not used by humans and encompasses non-productive and productive areas.

Non-productive areas and snow-covered areas without land use were classified based on the modeling results for the potential NPP (NPP_pot_) and allocated to cells where potential productivity was below 20 gC/m²/yr, following the approach by^33^.

Information on productive areas not or only rarely used by humans was derived from combining data depicting the human footprint, i.e. human pressures on the environment for 1993^63^ and annually from 2000 onwards^35^ with data on intact forest landscapes in 2000, 2013, 2016 and 2020 identified by^36^, downloaded from^64^. Both datasets were resampled to the target resolution of 30 arcseconds in ArcGIS Pro 3.2.2, linearly interpolated between data points and kept constant before the first, and after the last data point.

The human footprint values range from zero to 50, based on information on the extent of infrastructure, population density and agriculture. To identify unused areas, we only considered areas with a score of zero, i.e. areas with no discernable human presence. Since two different data sources were used for 1993 (data from^63^) and for 2000 onwards (data from^35,65^) we established continuity from 1993 to 2000 and beyond by adapting the 1993 layer. We first created the average of 1993 and 2000 and only counted cells with a value below 1 as wilderness and further set cells to zero that also have a value of zero in 2000.

Through the combination of both datasets, we differentiated between core wilderness and peripheral wilderness. Core wilderness was assumed to be without land use, while peripheral wilderness might be subject to infrequent land uses such as extensive, sporadic wood fuel gathering, mushroom picking, wild-honey-collecting, etc. Within the forest zone extent provided by^64^ core wilderness grid cells were defined as those, where both the human footprint was zero and an intact forest landscape was present. Outside the forest zone a human footprint score of zero was the only criterium for assigning core wilderness. We defined peripheral wilderness as those areas within the forest zone extent where only one of the two datasets indicated wilderness and also all areas remaining in cells where core wilderness covers only a fraction of the cell.

#### Grazing land, open wooded land and forestry

We classified several ESA CCI LC classes as being potentially used as permanent pastures and meadows (see Table 3), with decreasing suitability from class1 to class3 and distributed the national total given by FAO consecutively across these classes in the same procedure as for cropland. Since we assumed that some of these classes can potentially be used as both cropland and/or grazing land, the areas already assigned to cropland were subtracted from the areas available for grazing land in each step. Additionally, we re-classified peripheral wilderness areas to grazing land, in the rare cases where the FAO target value could not be met within suitability classes 1 to 3.

ESA CCI LC classes with tree cover between 15% and 40% were aggregated to “open wooded lands”. Following the approach that areas which are not unambiguously assigned to other land uses might also be used for grazing^33^, we included these open wooded lands in the grazing land-use type, but also allowed wood extraction through wood fuel collection.

We hence differentiated grazing land into two classes: grazing class “notrees” consists of grazing areas with grazing suitability 1, which were identified as agricultural areas or grassland by ESA CCI LC and have (almost) no trees. The remaining grazing land is categorized as grazing class “owl” (open wooded land), where woody vegetation is present and can also be harvested and the human influence on the landscape is less than in grazing class “notrees”.

Land predominantly used for forestry was mapped based on ESA CCI classes of closed forests (>40% tree cover based on the plant functional types given in the ESA CCI LC product description). We differentiate coniferous and non-coniferous forests.

### Land-use intensity: biomass harvest, harvest residues, and land-use induced changes in NPP

Based on the land-use area dataset all components of the framework of Human Appropriation of Net Primary Production (HANPP) were calculated using adapted methods from^1,37,51,66^. This framework discerns three different types of NPP: potential NPP (NPP_pot_), i.e., the NPP that would prevail in the absence of land use but with current climate; the NPP of the actually prevailing vegetation (NPP_act_), and the NPP that remains intact in ecosystems after harvest (NPP_eco_). The difference between NPP_pot_ and NPP_act_ is the human-induced change in NPP due to land conversions (HANPP_luc_); the difference between NPP_act_ and NPP_eco_ is HANPP_harv_, i.e., the biomass extracted or killed during harvest. HANPP is the sum of HANPP_luc_ and HANPP_harv_, equals the difference between NPP_pot_ and NPP_eco_, and integrates output intensity and system-level intensity. HANPP can also be expressed as fraction of NPP_pot_, then we denote it as HANPP%.

#### NPP_pot_ of all land-use classes

NPP_pot_, the potential productivity in the absence of human land use, is the reference point of the HANPP framework and was derived from the Dynamic Global Vegetation Model (DGVM) LPJ-GUESS^67^ in a run that assumed no land use or land management but used historical climate forcing data from 1900–2015 as in the preceding global HANPP study by^1^. We set negative NPP values to zero and calculated 5-year moving averages to reduce the influence of annual fluctuations.

The computational requirements of such a global model run currently limit the feasible spatial resolution to 30 arcminutes, which is a lot coarser than the 30 arcsecond resolution aimed for in the LUIcube. While previous studies have resolved this mismatch in resolution with simple bilinear interpolation^1^, we applied an alternative approach that takes fine-scale local conditions and their influence on NPP into account. This approach downscales the coarse-scale DGVM results based on NPP patterns derived from the empirical MIAMI model^68^ utilizing data on temperature and precipitation available at 30 arcseconds^69^ while retaining the original NPP totals on the level of ecoregions. The approach is described in detail in^70^.

#### HANPP on built-up land

On built-up land, which is dominated by unproductive, sealed areas, but also includes urban green spaces such as parks and gardens, HANPP_harv_ was estimated at 1/6^th^ of NPP_pot_ and HANPP_luc_ was estimated at 2/3^rd^ of NPP_pot_^1^.

#### HANPP on cropland

The national level of HANPP_harv_ per crop is calculated based on the crop production reported in FAOstat^34^ (downloaded on 10^th^ June of 2024) and factors to account for losses and byproducts accruing during harvest^51,66^. The spatial distribution of HANPP_harv_ within a country follows the pattern of production derived from the SPAM database. For cells not considered as cropland in the SPAM database, but classified as cropland in the LUIcube, we assumed the yield to be at the lower end of the range within a country as to not overemphasize the production in these grid cells, and set it to 80% of the 5^th^ percentile to ensure also a reduced yield in cases where one yield level is given for the whole country. If the SPAM database did not provide information on the production of a given crop in a country the global median yield of this crop was applied. A moving average was calculated to smooth out the edges of the 5-arcminute resolution of the SPAM layers.

NPP_act_ was then calculated by accounting for pre-harvest losses (as in^1^) and compared to the NPP_pot_ on the respective cropland area per cell to arrive at HANPP_luc_.

#### HANPP of industrial roundwood and wood fuel harvest

As described in^1,51^ factors were used to derive HANPP_harv_ (the sum of all biomass felled, i.e. stem wood, bark, branches, foliage, understory, roots etc.) from the national level wood harvest statistics from FAOstat^34^ (downloaded on 10^th^ June of 2024), which reports roundwood volumes (excluding bark). NPP_act_ of woodlands was assumed to be equal to NPP_pot_, i.e. HANPP_luc_ is assumed to be zero^32,43^.

The LUIcube offers a finer thematic resolution than previous publications on HANPP, differentiating between industrial roundwood (IR) and wood fuel (WF) harvest as well as between coniferous and non-coniferous harvest and introducing open wooded lands as parts of grazing land where also wood extraction takes place.

The procedure for allocating national data to the grid worked as follows: IR harvest was assumed to predominantly occur in closed forests designated for forestry, while WF harvest was assigned to closed forests and open wooded lands proportionally to NPP_act_. As in previous publications, the pattern of harvest was assumed to follow NPP_act_ patterns and the limit for wood harvest was set to 70% of NPP_act_^1,37^.

**Fig. 8:**
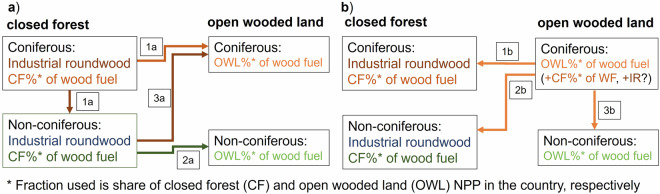
Allocation procedure for wood harvest, exemplified for coniferous tree type. The procedure starts with closed forests (**a**) and then proceeds to open wooded lands (**b**).

First, harvest occurring in closed forests was allocated, starting with the allocation of IR harvest to the grid. In countries where the IR harvest exceeded the available NPP_act_ of the respective tree type (i.e., coniferous or non-coniferous, hereafter termed tree type1 for illustrative purposes), the WF harvest was moved to open wooded lands of the same tree type1. Any still exceeding IR harvest was allocated to the closed forests of the other tree type2 (Fig. 8a, step 1a), as the tree type classification is not assumed to be 100% accurate. If the closed forest of the other tree type2 could not provide enough NPP_act_ for IR and WF harvest, the assigned WF harvest was moved to open wooded lands of tree type2 first (step 2a). Then, the remaining exceeding IR harvest was added to the harvest allocated to open wooded lands of tree type1 (step 3a).

Second, the harvest in open wooded lands was allocated. If the assigned WF harvest exceeded the NPP_act_ available in open wooded lands of the respective tree type1, it was re-allocated to be harvested in closed forests of the same tree type1, to the extent NPP was still available for harvest (step 1b). Any remaining WF harvest was then allocated to closed forests of the other tree type2 (step 2b). If the available NPP_act_ in these classes could still not cover the harvest, as would be the case if steps 1-3a had been triggered, it was moved to open wooded lands of the other tree type2 (step 3b), and was reduced if this was not sufficient.

#### HANPP of livestock grazing

The national level livestock feed demand was calculated as a “grazing gap”, i.e. the difference between livestock feed demand and the sum of feed crops and crop residues used as feed, as described in^51,66^. The feed demand of roughage consuming livestock was calculated based on the factors and linear regressions between average daily feed intake per head and average national milk yield and carcass weight from^71^. However, we here defined the feed demand of cows and cattle as the minimum between the estimate for milk cows and beef cattle, as opposed to previous estimates which used the maximum^1,37,51,72^, to increase the consistency with other estimates described in^73^. National amounts of feed supply from market feed crops were sourced from Supply Utilization Accounts provided by FAO^34^. Supply Utilization Accounts of by-products from oil production (oil cakes, e.g., soybean cake) are currently not reported by FAO and hence were estimated: the production of oil was extrapolated from vegetable oil production via technical conversion factors. Total imports and exports of oilcakes for each country were estimated by aggregating the bilateral trade data of the FAO (prioritizing importer reported before exporter reported values, see^74^). The supply of oilcakes was calculated as production plus imports minus exports and was entirely attributed to animal feed. The use of crop residues as feed was included as in^51^ but adjusted by introducing thresholds to ensure that minimum 30% of the total feed demand is covered by roughage (crop residues and grazing) and minimum 15% by grazing alone.

NPP_act_ on grazing lands was estimated by taking NPP_pot_ as a starting point and reducing it in the case of grazing-induced degradation^75^, and in the case of productivity changes due to ecosystem conversions, i.e. the replacement of forests by pastures and meadows. The portion of NPP_act_ available for grazing was calculated based on data on the proportion of non-palatable woody and belowground biomass^76^. Boosting of NPP due to fertilization was allowed in countries which applied more than 5% of their overall fertilizer use to grasslands each year^77^ and only assumed to occur on the grazing land class “notrees”.

The grazing mapping algorithm from the national level to the grid was adapted in this study. While previously grazing HANPP_harv_ was assigned to the grid based on NPP_pot_ patterns and the assumption that grazing intensity is disproportionally higher on high-productivity grazing lands, this study integrates human population density and the grazing land classification as additional factors to achieve a clearer separation between intensively used pastures and extensively used, remote rangelands.

**Fig. 9:**
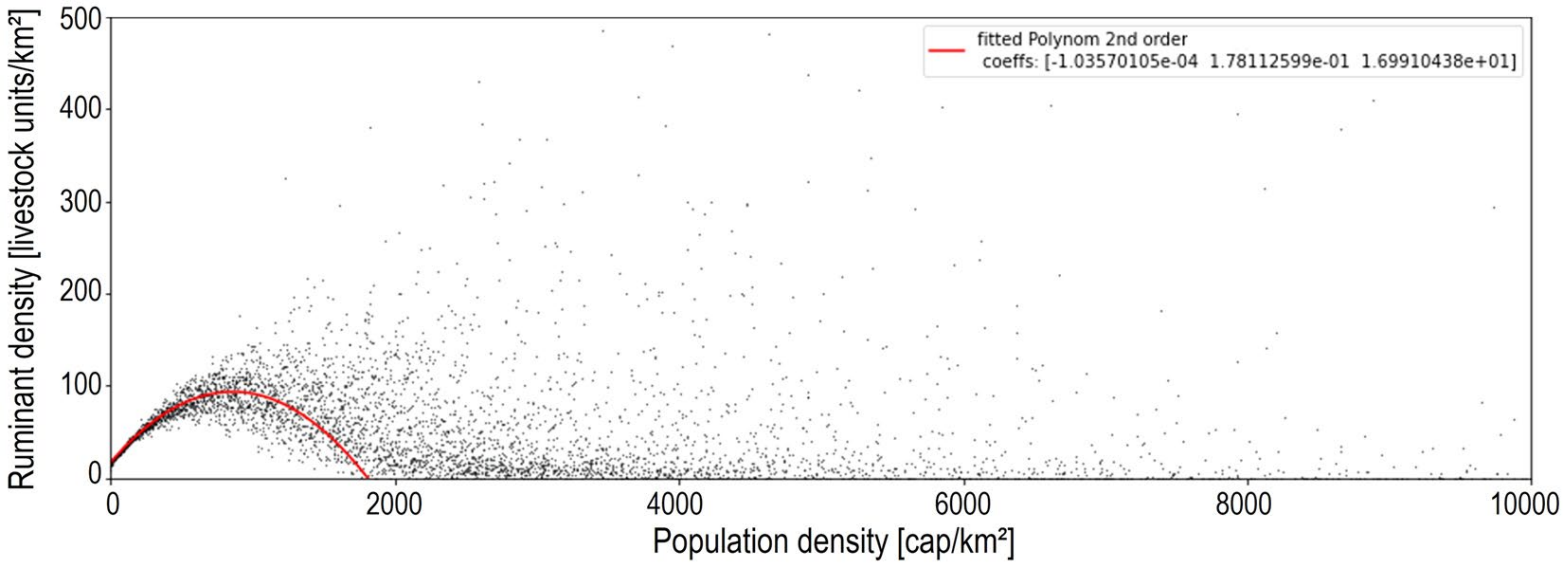
Plot of grid-cell values of population density VS. ruminant density with derived best-fitting polynomial function of 2^nd^ order.

The influence of population density on livestock distribution was derived from the Gridded Livestock of the World dataset^78,79^ and the Global Human Settlement Layer^80^, by plotting ruminant density against population density and calculating coefficients of a 2^nd^ order polynomial function (Fig. 9).

**Fig. 10:**
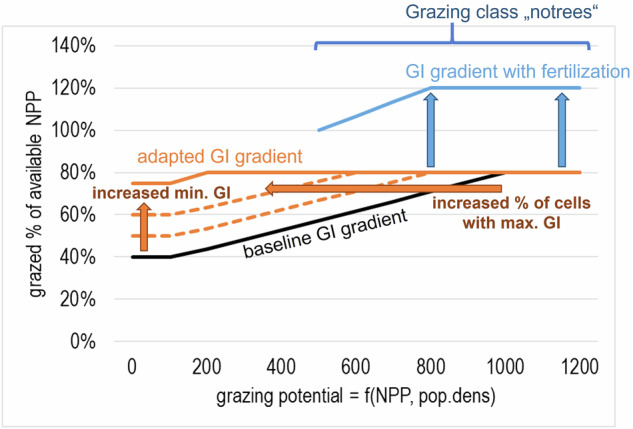
Grazing intensity (GI) gradient used to distribute grazing harvest per country. The baseline GI gradient (in black) was adapted by step-wise increasing the minimum GI and the percentage of cells with maximum GI (in orange). Boosting of NPP available for grazing on grazing land class “notrees” was allowed if fertilization was indicated (in blue).

The multiplication of NPP available for grazing and the influence of population density (based on the derived polynomial function) was used to assign a potential for grazing to each grid cell. Within the national distribution of “grazing potentials” we assigned a minimum grazing intensity of 40% of NPP to the 10% of cells with the lowest grazing potential, a maximum grazing intensity of 80% of NPP (due to seasonal constraints of productivity^76^) to the 15% of cells with the highest grazing potential, and linearly interpolated the grazing intensity in-between, creating the baseline grazing intensity gradient (see Fig. 10). If the biomass grazable under these intensity constraints was sufficient to cover the calculated national grazing demand, the factor derived from the ratio between biomass demand and availability (below 1) was applied to all grazing land cells to arrive at the actually grazed biomass per cell. If the grazing demand could not be covered with the baseline grazing intensity gradient, it was adapted: the minimum grazing intensity and the percentage of cells to which the maximum grazing intensity was allocated were increased at the same time until the grazing demand could be met or a minimum grazing intensity of 75% was reached and 85% of cells were assigned the maximum grazing intensity (see Fig. 10). This adaptation flattens the gradient along which grazing harvest is distributed among the grazing land cells within a country and increases the share of cells with a high grazing intensity. If the grazing demand could still not be met with this adaptation and no fertilization of grazing lands was indicated grazing harvest was reduced to the maximum level determined by these constraints.

For countries where increases of NPP_act_ on grazing land due to fertilization were indicated based on^77^, the grazing intensity gradient was adapted only to the extent to restrict the boosting of NPP required to meet the grazing demand to a factor below 2. NPP increases were only applied on grazing land class “notrees”. In extreme cases (e.g., India) when even the highest possible grazing intensity could not meet the calculated grazing demand grazable NPP on class1 grazing lands was boosted by a maximum factor of 3.

NPP_act_ on grazing lands of class “notrees” was subsequently increased by the additional NPP created through fertilization.

## Data Records

The LUIcube is available at Zenodo in the records listed in Table 5^81–89^.

**Table 5:** List of repository links for data download.

Zenodo repository name	Zenodo repository link	Content: LU classes
Dataset 1^81^	10.5281/zenodo.13990765	FO-con, FO-ncon, BU-builtup, WILD-core, WILD-periphery, WILD-nps
Dataset 2^82^	10.5281/zenodo.14000660	GL-owl
Dataset 3^83^	10.5281/zenodo.14013963	GL-notrees, CL-FALL, CL-OFIB
Dataset 4^84^	10.5281/zenodo.14011655	CL-WHEA, CL-MAIZ, CL-SOYB
Dataset 5^85^	10.5281/zenodo.14014550	CL-BARL, CL-MILL, CL-SORG, CL-RICE
Dataset 6^86^	10.5281/zenodo.14032829	CL-POTA, CL-SWPY, CL-REST
Dataset 7^87^	10.5281/zenodo.14035114	CL-CASS, CL-SUGC, CL-SUGB, CL-COTT, CL-VEFR
Dataset 8^88^	10.5281/zenodo.14035300	CL-BEAN, CL-OPUL, CL-BANP, CL-GROU
Dataset 9^89^	10.5281/zenodo.14035314	CL-OOIL, CL-FODD, CL-COFF

For each land-use class listed in Table 5 the LUIcube provides spatially explicit data layers of area and the associated NPP flows annually from 1992 to 2020 at 30 arcsecond resolution. Land-use classes are grouped into folders which include the data layers listed in Table 6 as GeoTIFF files, with file names following the structure year-data layer-unit-LU class (e.g., 1992area_km2_CL-WHEA.tif).

**Table 6:** List and description of land-use classes and data layers incorporated in the LUIcube.

LU type	LU class	LU class description	area [km²]	NPP_eco_ [tC/yr]	HANPP_luc_ [tC/yr]	HANPP_harv_ [tC/yr]
Cropland	CL-WHEA	wheat	x	x	x	x
Cropland	CL-RICE	rice	x	x	x	x
Cropland	CL-BARL	barley	x	x	x	x
Cropland	CL-MAIZ	maize	x	x	x	x
Cropland	CL-REST	rest of crops	x	x	x	x
Cropland	CL-MILL	millet	x	x	x	x
Cropland	CL-SORG	sorghum	x	x	x	x
Cropland	CL-POTA	potato	x	x	x	x
Cropland	CL-SWPY	sweet potato and yam	x	x	x	x
Cropland	CL-CASS	cassava	x	x	x	x
Cropland	CL-SUGC	sugarcane	x	x	x	x
Cropland	CL-SUGB	sugarbeet	x	x	x	x
Cropland	CL-BEAN	bean	x	x	x	x
Cropland	CL-OPUL	other pulses	x	x	x	x
Cropland	CL-SOYB	soybean	x	x	x	x
Cropland	CL-GROU	groundnut	x	x	x	x
Cropland	CL-OOIL	other oilcrops	x	x	x	x
Cropland	CL-COTT	cotton	x	x	x	x
Cropland	CL-BANP	banana / plantain	x	x	x	x
Cropland	CL-COFF	coffee	x	x	x	x
Cropland	CL-VEFR	vegetables / fruits	x	x	x	x
Cropland	CL-OFIB	other fibres	x	x	x	x
Cropland	CL-FODD	fodder crops	x	x	x	x
Cropland	CL-FALL	fallow	x	x	x	no harvest
Built-up land	BU-builtup	settlements, urban areas and infrastructure	x	x	x	x
Grazing land	GL-notrees	grazing land with (almost) no trees	x	x	x	x
Grazing land	GL-owl	grazing land characterized by open wooded lands	x	x	x	separate for grazing, wood (coniferous and non-coniferous)
Forestry	FO-con	forestry areas, mainly coniferous	x	x	= zero	x
Forestry	FO-ncon	forestry areas, mainly non-coniferous	x	x	= zero	x
Wilderness	WILD-core	unused productive wilderness areas	x	x	= zero	no harvest
Wilderness	WILD-periphery	productive wilderness areas that are sporadically used at very low intensity	x	x	= zero	no harvest
Wilderness	WILD-nps	unused unproductive wilderness areas	x	x	= zero	no harvest

## Technical Validation

The presented dataset builds on established methods and sources used to quantify and map land-use extent and intensity, but does so at a higher spatial and temporal resolution and for more specific land-use classes than before. The aggregated results and patterns are in line with the most comparable, preceding study by^1^ both in terms of absolute values (in tC/yr) and in terms of relative values (in % of NPP_pot_). The adaptations in methods resulted in some deviation for the results per land-use type, due to differences in area account (e.g. for built-up land), calculation procedure (e.g. reduction of feed demand) and increased thematic and spatial resolution. As discussed in^1^, the validity and robustness of the results is directly dependent on the input datasets, with the land-use area dataset and the NPP_pot_ data as the most influential factors.

The integrated land cover product from ESA CCI was validated against independent ground-based reference data and alternative estimates and claims an accuracy level of over 70%, with user accuracy values for cropland of ca. 90%^9^.

The SPAM database was validated with field-level data, feedback of local experts and against subnational statistical data not used for the model^20^. Although considerable discrepancies between global cropping system models exist^90^ SPAM offers the unique advantage to be available for numerous points in time, aiding the construction of a time series.

The productivity of the potential vegetation in the absence of human land use can theoretically only be validated in areas without human influence. On a global level, NPP_pot_ is derived with models, with complex, processed-based Dynamic Global Vegetation Models (DGVMs) representing the current state of the art. NPP_pot_ results of models show quite some variation in pattern and global totals, ranging from standard levels around 60GtC/yr^91–93^ (59.8GtC/yr for LPJ-GUESS, without nitrogen limitation^1^; 60.8GtC/yr for LPJ-GUESS with nitrogen limitation^1^, 62.1GtC/yr for LPJ-mL^51^), to outliers like 78.2GtC/yr (JS-BACH)^1^ in 2000. The LPJ-GUESS model (without nitrogen limitation) was selected in this study to ensure comparability with the run that emerged as the best-guess estimate in the preceding study^1^.

Where available, official statistical information from FAO and FRA, the prime agencies collecting relevant data on agriculture and forestry globally, was used. The LUIcube therefore captures global land-use based on widely used, best available data at the time.

Technical validity of the LUIcube was further ensured by checking and confirming consistency between country-level totals before and after each mapping algorithm was applied. This relates to country-level records of the area of cropland as well as permanent meadows and pastures, and agricultural and forestry production statistics. Gaps in the time series data from FAO and other input data were resolved as described under Methods to preclude missing values in the resulting data layers and consistency of value ranges with previous HANPP assessments is given.

## Usage Notes

The LUIcube provides data layers for area, NPP_eco_, HANPP_harv_ and HANPP_luc_ per land-use class annually from 1992 to 2020. The layers of land-use areas are provided in square kilometers (km²) per grid cell. All NPP flows are provided in tC/yr per grid cell. Adding HANPP_harv_ to NPP_eco_ results in NPP_act_, and adding HANPP_luc_ to NPP_act_ results in NPP_pot_ for the given land-use class. Area-intensive values (in gC/m²/yr) can be calculated by dividing the NPP flows by the area of the respective land-use class. HANPP in % of NPP_pot_ can be calculated by summing up HANPP_harv_ and HANPP_luc_ and dividing it by NPP_pot_. Areas and NPP flows of land-use classes can be aggregated to calculate their overall HANPP.

## Data Availability

The datasets presented in this study were produced with multiple MATLAB (R2021.a) and Python (3.8.10) scripts that are available at 10.5281/zenodo.14222929^94^.
